# Leaching of Different Clear Aligner Systems: An In Vitro Study

**DOI:** 10.3390/dj10020027

**Published:** 2022-02-14

**Authors:** Aseel Alhendi, Rita Khounganian, Abdullazez Almudhi, Syed Rizwan Ahamad

**Affiliations:** 1 Department of Pediatric Dentistry and Orthodontics, College of Dentistry, King Saud University, P.O. Box 60169, Riyadh 11545, Saudi Arabia; abalmudhi@ksu.edu.sa; 2 Department of Oral Medicine and Diagnostic Sciences, College of Dentistry, King Saud University, Riyadh 11545, Saudi Arabia; ritak@ksu.edu.sa; 3 Central Laboratory, Department of Pharmaceutical Chemistry, College of Pharmacy, King Saud University, Riyadh 11451, Saudi Arabia; srahamad@ksu.edu.sa

**Keywords:** clear aligners, leaching, chemical compound, benzene, gas chromatography–mass spectrometry

## Abstract

The aim of this study was to investigate and compare the leaching of four different clear aligner systems (Invisalign^®^, Eon^®^, SureSmile^®^, and Clarity^®^). Three sets of aligners as obtained from the four manufacturers were cut and immersed in glass vials containing ethanol with different solutions. The first was 100% ethanol, the second was 75% ethanol to 25% water, the third was 50% ethanol to water, the fourth was 25% ethanol to 75% water, and the last was 100% water. The samples were incubated for two weeks at 37 °C. Leached substances were detected by the gas chromatography–mass spectrometry (GC-MS). Eleven different chemical compounds were detected and confirmed. Benzene1,3-bis(1,1-dimethylethyl) was the only compound detected in all four systems at levels of 100% and 75% ethanol. Statistically, insignificant differences were detected among the different systems where leaching was confirmed. Eon^®^ system was the only material to show statistically significant differences when comparing the number of leached substances among the immersion solution concentrations. The four included systems showed variable degrees of leaching. The lowest amount of leached chemicals was observed in relation to the Invisalign^®^ system, while the highest number was found in the Eon^®^ system. None of the included clear aligner systems leached detectable amounts of bisphenol-A (BPA).

## 1. Introduction

Leaching is the extraction of a substance from a solid object or material by percolation. By investigating the leaching of a material into body fluids, the biocompatibility of that material is evaluated [1]. The oral environment is considered unique due to the presence of bacteria and bacterial byproducts as well as salivary enzymes that could contribute to the degradation of materials that come into contact with oral tissues. Previous investigations have concluded that water is considered a plasticizer of polymeric products through the weakening of intermolecular forces and subsequent chemical degradation. Furthermore, the degradation of plastics is accelerated by higher temperatures, mechanical wear, and the presence of enzymes [2]. 

Clear aligner therapy has evolved during the last decade, with the introduction of several new systems produced by many companies in the market [3]. Various clear aligner systems exist and have been internationally used by several professionals. Align Technology is considered the leading company in the clear aligner market, producing Invisalign^®^ (Invisalign; Align Technology, Santa Clara, CA, USA) system using SmartTrack^®^ material [4]. Meanwhile, Eon^®^ Holdings, established in 2011, designs and manufactures clear removable aligners using medical-grade polyurethane [5]. In 2018, 3M^™^ launched their own clear aligner system called Clarity^®^, made with durable and virtually invisible material [6]. Recently, SureSmile^®^ aligners were designed by Dentsply-Sirona in 2019. They are produced from Essix^®^ plastic, a thermoformed polyurethane material [7]. To our knowledge, with the exception of the Invisalign^®^ appliance, the leaching of clear aligners has not been fully investigated [8]. Given the similarity in composition and properties of acrylic resin employed in dentistry to fabricate dentures and/or removable orthodontic retainers to the materials used for clear aligners’ production, previously reported biological risks regarding those materials must be considered. Several investigators have concluded that the acrylic resin used to construct dentures could cause adverse side effects to oral tissues attributed to the leaching of residual monomers from polymeric materials [9]. The oral cavity is considered a unique environment where materials are exposed to thermal and pH changes, mechanical wear, and intraoral bacterial and salivary enzymatic degradation, which in turn promote leaching into the saliva [10,11,12].

Various types of orthodontic polymers are commercially available, such as polycarbonate, polyurethane, polyethylene, polyamides, and polymethyl methacrylate [13]. The basic constituent polymeric component of transparent trays, including clear aligners, is polyurethane, which is not an inert material [8]. A polymer is composed of a chain of organic units joined with urethane links [14]. One disadvantage of using synthetic polymers is the leaching of the residual monomers into the oral cavity saliva and consequently causing adverse biological reactions to living tissues [15]. In 2016, Thavarajah and Thennukonda [16] speculated that leached toxins from these devices might have the cumulative effect of causing allergic, anaphylactic, or nonspecific reactions. Leaching concentrations differ based on several factors related to monomer concentration, polymerization technique, and storage time. However, when the concentrations of the leached substances are high enough, potential risks, such as inflammation, irritation, or allergic reactions to the contacting tissues, may occur [9,17].

Plastic product toxicity can be caused by material degradation, additives, adsorbed contaminants, or the polymer matrix. Furthermore, the polymerization reaction itself could contribute to toxicity. Incomplete polymerization results in residual monomers, oligomers, low-molecular-weight polymeric fragments, catalysts, and solvents or internal chemicals that are incorporated during the plastic production process, such as bisphenol-A (BPA) [18]. BPA is a synthetic chemical compound that draws medical and dental professionals’ attention because of its biological hazards. In 2010, the World Health Organization released a full report about the toxicity of BPA. The benefit of BPA usage through adding it to polymers is to increase strength and transparency, which are important factors in appliance fabrication [19,20].

The aim of this study was to investigate and compare the leaching of four different clear aligner systems (Invisalign^®^, Eon^®^, SureSmile^®^, and Clarity^®^) using the gas chromatography–mass spectrometry unit (GC-MS). Our null hypothesis: There is no difference in leaching among the four clear aligner materials investigated. 

## 2. Materials and Methods

Three sets of aligners (maxillary and mandibular trays) were obtained from four different manufacturers (Invisalign^®^, Eon^®^, SureSmile^®^, and Clarity^®^) and accordingly were all cut into 5 × 5 mm squares. Each of them was immersed in separate glass sample vials containing alcoholic media to accelerate the degradation of the sample. Each material was soaked in five different solutions as follows: the first was absolute ethanol (100%), the second was 75% ethanol to 25% water, the third was 50% ethanol to water, the fourth was 25% ethanol to 75% water, and finally, 100% water solution served as the control. The samples were incubated for two weeks at 37 °C and shaken for five minutes at 150 rpm every day to simulate the accelerated aging process. 

The leached substances in the immersion medium were analyzed with a gas chromatography–mass spectrometry unit. An Agilent GC 7890A combined with a triple axis detector 5975 C single quadrupole mass spectrometer was used for GC-MS analysis. The chromatographic column was an Agilent HP 5MS column (30 m × 0.25 mm × 0.25 µm film thickness), with high-purity helium as the gas carrier, at a flow rate of 1 mL/min. The injector temperature was 280 °C, and it was equipped with a splitless injector at 20:1. The source temperature of MS was set at 230 °C, and the quad temperature was at 150 °C. The oven temperature was initially 50 °C (held for 1 min), then it was increased to 150 °C at 25 °C min^−1^ (held for 1 min), then it was further increased to 300 °C at 25 °C min^−1^ for 1 min. The scan range was set at 40–600 mass ranges at 70 eV electron energy and a solvent delay of three minutes. Finally, unknown compounds were identified by comparing the spectra with those of the NIST 2008 (National Institute of Standard and Technology library) [21]. The total time required for analyzing a single sample was 13 min. This study was approved by the Institutional Review Board, College of Medicine, King Saud University #E-20-4759 and CDRC #PR0112.

### Statistical Analysis

Quantitative data obtained from the gas chromatography analyses of samples from all groups at different concentrations were tabulated and analyzed using the Statistical Package for the Social Sciences (SPSS) software version 26.0 (IBM Inc., Chicago, IL, USA). Descriptive statistics (means, standard deviations, and frequencies) were used to express all quantitative variables. Two-way and one-way analysis of variance (ANOVA) were used to assess the differences among and within the various systems (Invisalign^®^, Eon^®^, SureSmile^®^, and Clarity^®^) at different solution concentrations. All assessments were carried out by one examiner and repeated twice to confirm reproducibility and reliability. Results were considered statistically significant when *p* ≤ 0.05.

## 3. Results

**Figure 1:**
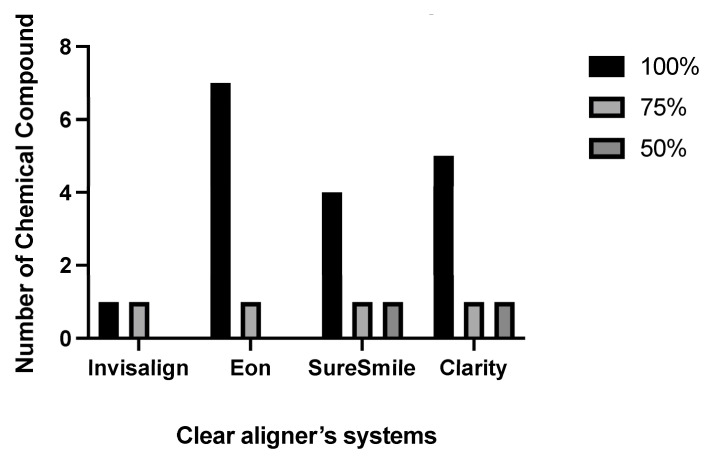
Number of leached chemicals among different systems at different concentrations.

Eleven different chemical compounds were detected and confirmed via the GC-MS library (Table 1). These chemicals were mainly presented in 100% ethanol in the different systems. The 75% and 50% ethanol concentrations had a single leached material. On the other hand, no leaching was observed at 25% ethanol and 0% (water), which served as the control. The highest number of chemicals was seen in the Eon^®^ system (seven compounds at two immersion concentrations), followed by Clarity^®^ (six compounds at three immersion concentrations), and then SureSmile^®^ (five compounds at three immersion concentrations). The Invisalign^®^ system had the least number of leached chemicals, presenting the same chemical compound at two immersion concentrations, as shown in Table 1 and Figure 1. 

**Figure 2:**
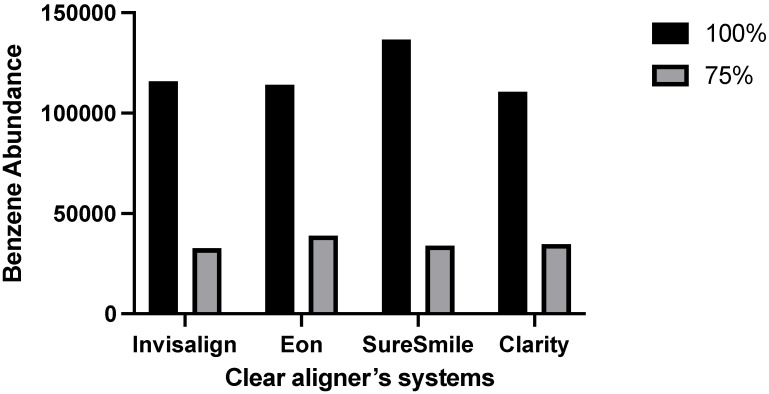
Abundance of leached benzene among different systems at different concentrations.

Benzene1,3-bis(1,1-dimethylethyl) was the only compound detected in all four systems at the levels of 100% and 75% ethanol. The 50% ethanol presented Phenol, 3,5-bis(1,1-dimethylethyl), in the SureSmile^®^ and Clarity^®^ systems with high percentages of 95% and 94%, respectively (Table 1). The abundance of leached benzene was detected at higher levels, at 100% ethanol compared to 75%, with all systems reporting similar amounts. SureSmile^®^ was the highest system with regard to benzene abundance at 100%, while Eon^®^ was the highest at 75% (Figure 2).

Leaching was confirmed from the different systems at variable immersion solution concentrations (Table 2). Furthermore, only the Eon^®^ system showed statistically significant differences when comparing the number of leached substances among the immersion solution concentrations (Table 3). Seven different leached chemical compounds were observed in a 100% immersion solution of the Eon^®^ system compared to a single material in 75% (benzene), and no leaching was observed in 50% ethanol. The number of leached chemicals was statistically insignificant in Invisalign^®^, SureSmile^®^, and Clarity^®^, regardless of the immersion solution concentration. No traces of BPA were detected among all the studied clear aligner systems.

## 4. Discussion

The assessment of the leaching of a material is vital since it is directly linked to the material’s toxicity and inversely related to the material’s safety and biological side effects. This experiment investigated the leaching of clear aligners manufactured by several systems (Invisalign^®^, Eon^®^, SureSmile^®^, and Clarity^®^). Eleven different chemical compounds were detected and confirmed using the GC-MS library. The main chemical compound identified in all systems at two levels of immersion solution concentrations (100% and 75% ethanol) was benzene, 1,3-bis(1,1-dimethylethyl). This compound was not detected when the alcohol concentration dropped to 50% or below. As illustrated in Table 1 and Figure 1, most of the chemical compounds were leached in the 100% ethanol concentration. The 75% ethanol solution showed benzene leaching from all systems, whereas 50% ethanol presented phenol, 3,5-bis(1,1-dimethylethyl), in the SureSmile^®^ and Clarity^®^ systems. Absolute water (0% ethanol) and 25% ethanol showed no leaching of any substance, indicating that reducing the alcohol concentration below 50% disables the solution from sample degradation. In contrast to our findings, a previous study in 2004 reported no leaching from Invisalign^®^ appliances. They concluded that the tested immersion solution of 75% ethanol showed no residual monomers or oxidate byproducts [8]. The other three systems were not previously evaluated with regard to leaching. A more recent investigation in 2016 reported that their tested clear aligner systems showed leaching of residual monomers in a 75% ethanol immersion solution, as this was the only concentration to be tested. They additionally concluded that different manufactured thermoplastic sheets presented different amounts of leached substances, which were inversely related to their biocompatibility [22]. 

In the present research, benzene was the only chemical compound detected in all systems in both 100% and 75% immersion solutions, which is a common chemical used to fabricate plastic products. The harmful effects of benzene are known and reported by the Centers for Disease Control and Prevention (CDC). The biological effect of this chemical ranges from skin rashes to irregular heartbeats, depending on the amount and exposure type [23]. The toxicity level of benzene as a degradation product varies depending on the polymer itself. For instance, benzene is considered to have high or medium toxicity in relation to polyamide and polyvinyl chloride polymers, respectively [18].

Few reported results have been found in the literature regarding detected chemical compounds in relation to clear aligners. Nevertheless, from a chemical point of view, some of the detected compounds are classified as metabolites. For instance, undecane, 4,6-dimethyl, has a biological role as a human metabolite. Heptadecane, 2,6,10,14-tetramethyl, is a metabolite observed in cancer metabolism, and octane, 3,5-dimethyl, also has a role as a human metabolite in addition to cancer metabolism. Dodecanoic acid ethyl ester, has likewise been classified as a metabolite [24,25]. All these metabolites were detected in relation to the Eon system^®^. On the other hand, multiple detected chemicals have reported biological hazards, such as nonadecane, which is mostly found in essential oils isolated from Artemisia armeniaca [26]. The Globally Harmonized System (GHS) of the Classification and Labeling of Chemicals has classified nonadecane as a danger aspiration hazard that might be fatal if it is swallowed or if it enters the airways [24]. 1-Octadecanesulphonyl chloride was detected in SureSmile^®^ at 100% ethanol, whereas phenol, 3,5-bis(1,1-dimethylethyl) was the only detected substance at 50% ethanol in both SureSmile^®^ and Clarity^®^. These are classified by the GHS as dangerous skin corrosion and irritation hazards, as they can cause skin burns and eye damage. All the reported side effects depend on multiple factors, such as the route of administration, concentration of the substance length of contact, and others [24,25]. Phenol, 2,4-bis (1,1-dimethylethyl) has a role as a bacterial metabolite, an antioxidant, and a marine metabolite; it is an alkylbenzene and a member of the phenols group. The GHS classified this chemical as a health and environmental hazard. It is capable of producing organ toxicity, skin irritation, and eye damage based on the route of administration [24,27]. Moreover, methoxyacetic acid, 2-tridecyl ester, is a phytochemical compound with reported cytotoxicity [28]. The toxicological properties of ether, hexyl pentyl, have not yet been fully investigated [29].

The laboratory settings followed in this experiment might not reflect the full picture of the potential degradation of clear aligners. This is due to other contributing intraoral factors that cannot be applied in vitro. Those appliances are subjected to mechanical abrasion, chemically caused attrition, temperature variation, and enzymes [8]. Not only do fabrication material and immersion solutions control the leaching process, but other polymer-related factors can play a role in leaching, including incomplete polymerization, molecular weight, and the density of a polymer [22,30]. The intraoral aging process differs from the in vitro conditions, as the former involves chemical and mechanical factors including salivary enzymes, bacterial byproducts, abrasion, and pH levels [12]. Further, the strength of the immersion solution plays a role in chemical leaching. Previous investigators utilized 75% alcoholic media, which is considered a potent immersion solution. The strength of the current investigation is the testing of higher and lower ethanol concentrations, where the lower levels of alcohol presented no chemical traces [8,22].

Although no traces of BPA were detected under the current investigation conditions, the safety of these appliances is debatable. It is well-established that the toxicity of a biomaterial is directly proportional to the number of leached compounds and the amount of each chemical [22]. Invisalign^®^ was found to be the safest among the tested systems, as it leached only one confirmed chemical compound (benzene). On the other hand, Eon^®^ was considered the least safe because seven chemicals were detected in this system. This was followed by Clarity^®^ and SureSmile^®^, presenting six and five detectable compounds, respectively, at three variable solution concentrations.

Fortunately, clear aligners, in contrast to other orthodontic appliances and/or materials, remain in direct contact with oral tissues for short periods of time (approximately 7–14 days) compared to comprehensive treatment or retention appliances. The constant manufacturers’ recommendation for patients is to change their aligners every one to two weeks. However, the cumulative effect of these appliances should not be underestimated, and future well-constructed clinical trials on patients undergoing clear aligner therapy should be conducted to evaluate the leaching of those appliances into body fluids (saliva and urine). 

## 5. Conclusions

All four included systems showed some degree of leaching in alcoholic immersion media. The 100% ethanol solution presented the most leached substances (11 chemical compounds). When the ethanol concentration was below 50%, no chemicals were detected. The least amount of leaching was observed in relation to the Invisalign^®^ system, with the same chemical compound detected at 100% and 75% ethanol (benzene, 1,3-bis(1,1-dimethylethyl)), while the highest number of leached materials was found in the Eon^®^ system, presenting seven different chemicals at both 100% and 75% ethanol. Under the current experimental conditions, none of the included clear aligner systems leached detectable amounts of BPA. 

## Figures and Tables

**Table 1:** Detected and confirmed chemical compounds and their concentrations in relation to clear aligners’ systems and immersion solution.

Immersion Solution Concentration	Chemical Compound	Substance Concentration %			
		Invisalign^®^	Eon^®^	Sure Smile^®^	Clarity^®^
100% Ethanol	Benzene, 1,3-bis(1,1-dimethylethyl)	42%	16.1%	37%	32%
	Phenol, 2,4-bis(1,1-dimethylethyl)	ND	11%	25%	16%
	Undecane, 4,6-dimethyl	ND	5.4%	ND	8%
	Heptadecane, 2,6,10,14-tetramethyl	ND	2.1%	6%	ND
	Octane, 3,5-dimethyl	ND	4.3%	ND	ND
	Nonadecane	ND	5.3%	ND	ND
	Dodecanoic acid, ethyl ester	ND	16%	ND	ND
	1-Octadecanesulphonyl chloride	ND	ND	8.1%	ND
	Methoxyacetic acid, 2-tridecyl ester	ND	ND	ND	8%
	Ether, hexyl pentyl	ND	ND	ND	7.6%
75% Ethanol	Benzene, 1,3-bis(1,1-dimethylethyl)	20.3%	74.2%	80%	58%
50% Ethanol	Phenol, 3,5-bis(1,1-dimethylethyl)	ND	ND	95%	94%

ND: Non-detectable chemical compound.

**Table 2:** Comparison of different systems at variable immersion solution concentrations.

Immersion Solution Concentration	Aligner’s System	N	Mean	Std. Deviation	Std. Error	F	*p*-Value	95% Confidence Interval	
								Lower Bound	Upper Bound
100% Ethanol	Invisalign^®^	11	10,535.82	34,943.36	10,535.82	0.961	0.42	−12,939.45	34,011.08
	Eon^®^	11	39,547.09	43,964.78	13,255.88			10,011.15	69,083.03
	SureSmile^®^	11	24,809.27	46,290.84	13,957.21			−6289.34	55,907.88
	Clarity^®^	11	22,472.73	34,562.63	10,421.02			−746.76	45,692.22
75% Ethanol	Invisalign^®^	11	2981.27	9887.76	2981.27	0.006	0.999	−3661.42	9623.96
	Eon^®^	11	3548.27	11,768.29	3548.27			−4357.77	11,454.32
	SureSmile^®^	11	3093.64	10,260.43	3093.64			−3799.42	9986.69
	Clarity^®^	11	3164.82	10,496.51	3164.82			−3886.84	10,216.47
50% Ethanol	Invisalign^®^	11	0.00	0.00	0.00	0.667	0.577	0.00	0.00
	Eon^®^	11	0.00	0.00	0.00			0.00	0.00
	SureSmile^®^	11	3137.64	10,406.36	3137.64			−3853.45	10,128.73
	Clarity^®^	11	2966.91	9840.12	2966.91			−3643.78	9577.59

**Table 3:** Comparison between systems in relation to number of detected chemical compounds at variable immersion solution concentrations.

Aligner’s System	Immersion Solution Concentration		Mean Difference	Std. Error	F	*p*-Value	95% Confidence Interval	
							Lower Bound	Upper Bound
Invisalign^®^	100%	75%	7554.55	8940	0.738	0.487	−14,486	29,595
		50%	10,535.82	8940			−11,504	32,576
	75%	100%	−7554.55	8940			−29,595	14,486
		50%	2981.27	8940			−19,059	25,021
	50%	100%	−10,535.82	8940			−32,576	11,504
		75%	−2981.27	8940			−25,021	19,059
Eon^®^	100%	75%	35,998.81818 *	11204	7.627	0.002 *	8377	63,621
		50%	39,547.09091 *	11204			11,925	67,169
	75%	100%	−35,998.81818 *	11204			−63,621	−8377
		50%	3548.27	11204			−24,074	31,170
	50%	100%	−39,547.09091 *	11204			−67,169	−11,925
		75%	−3548.27	11,204			−3,1170	24,074
SureSmile^®^	100%	75%	21,715.64	11,950	2.197	0.129	−7745	51,177
		50%	21,671.64	11,950			−7789	51,133
	75%	100%	−21,715.64	11,950			−51,177	7745
		50%	−44.00	11,950			−29,505	29,417
	50%	100%	−21,671.64	11,950			−51,133	7789
		75%	44.00	11,950			−29,417	29,505
Clarity^®^	100%	75%	19,307.91	9217	2.956	0.067	−3413	42,029
		50%	19,505.82	9217			−3215	42,227
	75%	100%	−19,307.91	9217			−42,029	3413
		50%	197.91	9217			−22,523	22919
	50%	100%	−19,505.82	9217			−42,227	3215
		75%	0	9217			−22,919	22,523

*: Statistically significant < 0.05.

## Data Availability

The data supporting reported results analyzed or generated during the study are available upon request from the corresponding author.
